# The Maintenance of Memory Plasma Cells

**DOI:** 10.3389/fimmu.2019.00721

**Published:** 2019-04-05

**Authors:** Laleh Khodadadi, Qingyu Cheng, Andreas Radbruch, Falk Hiepe

**Affiliations:** ^1^Deutsches Rheuma-Forschungszentrum Berlin—A Leibniz Institute, Berlin, Germany; ^2^Charité – Universitätsmedizin Berlin, Corporate Member of Freie Universität Berlin, Humboldt-Universität zu Berlin, and Berlin Institute of Health, Campus Charité Mitte, Medizinische Klinik mit Schwerpunkt Rheumatologie und Klinische Immunologie, Berlin, Germany

**Keywords:** plasma cells, memory plasma cells, long-lived plasma cells, maintenance, survival, bone marrow, inflammation, autoreactivity

## Abstract

It is now well accepted that plasma cells can become long-lived (memory) plasma cells and secrete antibodies for months, years or a lifetime. However, the mechanisms involved in this process of humoral memory, which is crucial for both protective immunity and autoimmunity, still are not fully understood. This article will address a number of open questions. For example: Is longevity of plasma cells due to their intrinsic competence, extrinsic factors, or a combination of both? Which internal signals are involved in this process? What factors provide external support? What survival factors play a part in inflammation and autoreactive disease? Internal and external factors that contribute to the maintenance of memory long-lived plasma cells will be discussed. The aim is to provide useful additional information about the maintenance of protective and autoreactive memory plasma cells that will help researchers design effective vaccines for the induction of life-long protection against infectious diseases and to efficiently target pathogenic memory plasma cells.

## Historical Aspects of Memory Plasma Cells

The term “plasma cell” (PC) was introduced by the anatomist Wilhelm Waldeyer in 1875, but it is doubtful whether he was actually referring to the same cells now known as antibody-secreting plasma cells. In 1895, Marshalko described oval cells with a strong basophilic cytoplasm and an eccentric nucleus containing coarse heterochromatin, which indeed corresponds to the current morphological definition of plasma cell (1). Its role as an antibody-secreting cell (ASC) was first demonstrated by Astrid Fagraeus in 1947 (2). Max Cooper and Robert Good identified lymphocytes (later termed as B lymphocytes) in the bursa of Fabricius of chickens, which is equivalent to the human bone marrow as the precursor of plasma cells (3). The phenotype and isotype of plasma cells can differ based on the type of activated B cells (naïve or memory, lymph nodes or spleen, and B1 or B2) and stimuli (T-independent or T-dependent antigens) (4). Bone marrow (BM) plasma cells are the main source of circulating antibodies (5–8). Plasma cells are specialized to secrete large amounts of antibodies (about 10^3^ per second) (9, 10). For many years, the general opinion was that plasma cells are short-lived since they can only survive a few days under *in vitro* conditions Therefore, it was postulated that plasma cells are replenished via the constant activation of memory B lymphocytes (3, 11).

In 1997, Andreas Radbruch's group showed that antigen-specific plasma cells generated in ovalbumin (OVA)-immunized mice were maintained in the bone marrow for up to 120 days without proliferation (12, 13). At about the same time, Slifka et al., using an entirely different technical approach, demonstrated that plasma cells can persist in murine bone marrow for more than 1 year, even if their precursors were blocked (6). Recently, Hammarlund et al. observed the survival of antigen-specific plasma cells induced by vaccination in the bone marrow of rhesus macaques, a species with a lifespan similar to humans, for more than one decade in spite of sustained memory B cell depletion (14).

Plasma cells can be generally divided into two distinct categories based on their lifespan: (a) short-lived plasma cells/plasmablasts (proliferating cells with a life span of 3–5 days) and (b) long-lived plasma cells (non-proliferating cells with a life span of several months to lifetime). The term- antibody secreting cells (ASCs) refers to both short-lived and long-lived plasma cells. It is not fully understood whether long-lived plasma cells represent the final differentiation stage of short-lived plasma cells, or whether short- and long-lived plasma cells belong to completely separate plasma cell populations (15). While long-lived plasma cells are mainly formed during germinal center reaction secreting high-affinity class switched antibodies located in BM, short-lived plasma cells are mainly formed in extra-follicular sites of secondary lymphoid organs expressing low-affinity IgM antibodies (16, 17). The competence to become a long-lived plasma cell is distinct from the basic ability to become a plasma cell (18). It is presumed that not all plasma cells are long-lived *per se*. In our opinion, long-lived plasma cells fulfill the criteria of memory cells as they continuously secrete the antibodies independently of their precursor cells (B cells), T cell help and antigen presence. Therefore, we suggest to use the term “memory plasma cell.”

We have proposed that, in order to become a memory cell, short-lived plasmablasts need a special environment: the so-called plasma cell survival niche (19, 20). This survival niche is composed of cellular components and soluble factors derived from these cells. If migratory plasmablasts reach the survival niche and receive survival factors there, they will become memory plasma cells; otherwise, they will remain short-lived and die. The bone marrow is the primary niche for memory plasma cells. Of note, plasma cells can survive for decades in the hypoxic bone marrow, and it has been shown that hypoxic condition enhances the survival of human plasma cells *in vitro* (21). Therefore, the hypoxic environment could be one of the factors that contribute to the long-term survival of memory cells. The number of plasma cell survival niches in a given organ is limited. This, in turn, limits the number of memory plasma cells per organism (22). A recently introduced mathematical model provides a possibility to quantify the niche-related dynamics of plasma cells (23). However, the long half-life of plasma cells is a new area of exploration. Most of our current knowledge about memory plasma cells is from mouse models. However, we should also consider some differences between human and mice (24). There are many questions to be answered, for example, whether the internal trigger for transformation into memory plasma cells is the intrinsic program of plasma cells, or if it is related to external signals from the plasma cell survival niche.

## Extrinsic Survival Factors (Signals)

Extracellular factors can be divided into two general categories: cellular compartments and molecular compartments.

### Cellular Compartments

Cellular compartments of plasma cell survival niches are composed of stromal cells (key players) and originated hematopoietic cells (accessory cells).

#### Stromal Cells

Stromal cells are a complex network of various subpopulations, including fibroblasts, endothelial cells, fat cells, and reticular cells, almost all of which are bone marrow stromal cells of mesenchymal origin (25). They provide signals by secreting growth factors or by making direct cell-cell contacts needed for hematopoiesis (including the progression of B-lymphoid lineage cells) or for the survival of memory plasma cells (26, 27). *In vitro* studies show that co-culture of plasma cells with stromal cells significantly increases the life span of plasma cells (27). Reticular stromal cells, a minor subpopulation of stromal cells, express CXC-chemokine ligand 12 (CXCL12, a ligand of CXCR4 expressed on plasma cells) and are scattered throughout the bone marrow (28). It has been shown that high numbers of plasma cells are in contact with these CXCL12-expressing cells in CXCL12/GFP reporter mice (28). Furthermore, *in vivo* intravital microcopy studies have demonstrated that direct contacts form between plasma cells and reticular stromal cells, that reticular stromal cells form a static component of the plasma cell survival niche, and that about 80% of plasma cells directly contact reticular stromal cells in a non-random fashion (29). However, a recent study has shown that cell-cell contact is not necessary for the survival of human bone marrow plasma cells *in vitro* (21).

Fibroblasts form part of the survival niches for memory plasma cells in the bone marrow by producing IL-6 and CXCL12 (30, 31). Other evidence shows that fibroblasts from the lymph nodes (LN) and spleens of mice and humans can also promote plasma cell survival *in vitro* (27, 32). A new subset of fibroblastic reticular cells (FRCs) that form dense meshworks in the medullary cords of lymph nodes, where many plasma cells reside, has been recently identified. Medullary FRCs have also been described as major local producers of plasma cell survival factors IL-6, BAFF, CXCL12 and APRIL. “*In vitro*, medullary FRCs alone or in combination with macrophages promote plasma cell survival while other LN cell types do not have this property” (33).

Another hypothesis of how stromal cells and plasma cells communicate involves the release of extracellular vesicles from bone marrow-derived mesenchymal stromal cells (MSCs). This novel mechanism of cell-cell communication over short and long distances supports the concept of *ex vivo* survival of human antibody secreting cells (34).

#### Hematopoietic Niche Components

Hematopoietic niche components (HNC) such as megakaryocytes (35), basophils (36, 37), dendritic cells and monocytes/macrophages (38), myeloid progenitors (39), neutrophils (40), and eosinophils (41) act as accessory cells of the plasma cell niche. Hematopoietic cells are associated with memory plasma cells in bone marrow and support their survival mainly by secreting the survival factors APRIL (a proliferation-inducing ligand) and IL-6 (see below). Plasma cells express BCMA (B- cell maturation antigen) and IL-6R (IL-6 receptor), receptors for APRIL and IL-6, respectively. Among hematopoietic components, eosinophils are the best characterized cells as source of APRIL and IL-6 (42, 43). In accordance with the importance of eosinophils in maintaining memory plasma cells, animal studies have shown that the number of plasma cells is significantly reduced in the bone marrow and gastrointestinal tract of eosinophil-deficient ΔdblGATA1 mice (41, 43, 44). However, two more recent, independent studies suggest that eosinophils are not essential for plasma cell survival in the bone marrow (45, 46). This discrepancy might be due to the effects of different environmental factors, especially microbiota. Microbiota can play a role during early life and may thus influence the generation of plasma cells and total immunoglobulin concentrations in adult animals. A new study indicates that microbiota-specific IgA-producing gut plasma cells generated during infancy live for many decades (47). This suggests that signals from the microbiota can impact on plasma cell pools. However, eosinophils are not the only APRIL source that supports the survival of plasma cells. APRIL can be produced by a variety of other bone marrow cells (35, 48).

Considering the short life span of cells of hematopoietic origin compared to that of long-lived memory plasma cells, the maintenance of static memory plasma cells does not depend on a single cellular source of survival factors. The multicomponent plasma cell survival niche model suggests that different dynamic hematopoietic cells can compensate for the loss of a particular cell type (48). Regulatory T cells (Tregs)—the other type of cells of hematopoietic origin—also play a supportive role in the maintenance of memory plasma cells. It has been reported that the loss of T regulatory cells correlates with the reduction of memory plasma cell populations in the bone marrow. Although the mechanism for this remains unclear, the close association of Treg cells and plasma cells suggests that communication between these populations takes place through cell-cell contact or soluble factors (49). In the bone marrow Treg cells express high levels of Treg effector molecules CTLA-4, and that deletion of CTLA-4 results in elevated plasma cell numbers. These findings indicate a possible regulatory effect of CTLA-4 expression on Tregs, a population which acts on the plasma cell pool in the bone marrow (49). However, it is also known that plasma cell survival depends on constitutive signals through CD80/CD86, which is presented by CD11c cells under T regulatory cell control (49, 50).

### Molecular Niche Components

Molecular niche components (MNC) include soluble factors and membrane-bound factors.

#### Soluble Factors

Soluble factors that contribute to plasma cell survival are cytokines and chemokines. A wide variety of cytokines, including IL-6 and members of the tumor necrosis factor (TNF) superfamily (APRIL, BAFF, and TNF-alpha), play an important role. IL-6 binds to the IL-6 receptor expressed on plasma cells, enhances their survival and maintains antibody titers *in vitro* (51). However, plasma cell survival is not significantly decreased in IL-6-null mice (51). In human, IL-6 is mandatory for *in vitro* generation and survival of memory plasma cells in combination with either APRIL or stromal cell-soluble factors (52). APRIL (a proliferation-inducing ligand) and BAFF (B-cell activating factor) are also important factors for memory plasma cell maintenance (53). Structurally, APRIL and BAFF are very similar cytokines that belong to the TNF superfamily. Both can bind with high affinity to B-cell maturation antigen (BCMA) and to transmembrane activator and calcium-modulator and cyclophilin ligand interactor (TACI), which is expressed by B cells at various stages of maturation; however, only BAFF can react with the BAFF receptor (BAFF-R) (54). Plasma cells express BCMA and TACI, but only low levels of BAFF-R (55). APRIL, which competes with BAFF for receptor binding sites, is expressed by eosinophils (41), megakaryocytes (35) and myeloid-derived cells, including monocytes, macrophages, and dendritic cells (41, 54). APRIL and BAFF bind to BCMA (their shared receptor) and promote plasma cell survival by inducing the anti-apoptotic molecule Mcl-1 of the Bcl-2 family (see below) (56). Neutralization of BAFF and APRIL with TACI-Ig depletes plasma cells in the bone marrow, whereas the presence of either BAFF or APRIL alone is sufficient to sustain the plasma cell population (53, 57). Unlike IL-6 deficiency, BCMA deficiency has a great impact on the loss of memory plasma cells (57). TNF-alpha has also been shown to support the survival of human plasma cells *in vitro* (30, 51). Taken together, these data suggest that the TNF superfamily and IL-6 are essential for the long-term maintenance of plasma cells in the bone marrow.

Chemokines and their receptors are crucial for the control of lymphocyte trafficking. CXCL12 (also known as SDF1) and its receptor CXCR4 are important for the migration of plasmablasts to the bone marrow for final differentiation into plasma cells, and for the maintenance of effective humoral immunity (17, 58). CXCL-12 has two main effects on plasmablasts and memory plasma cells. First, it acts as a chemokine and guides the plasmablasts from secondary organs to the bone marrow (59) and, second, it acts as a survival factor for plasma cells (as was shown *in vitro*) (51, 60). CXCL12-expressing stromal cells guide plasmablasts (expressing CXCR4) toward unique environments rich in anti-apoptotic survival factors in the bone marrow for their survival (61). In humans, the migration of plasmablasts requires glucose oxidation, which is controlled by CXCL12/CXCR4-mediated activation of the protein kinase AKT (62). CXCL12 itself also promotes plasma cell survival in murine bone marrow *in vitro* and *in vivo* (17, 27). Hence, the chemokine CXCL12 promotes the entry of CXCR4-expressing plasma cells into the bone marrow and the long-term survival of plasma cells (42).

#### Membrane Bound Factors: Memory Plasma Cell Surface Markers and Adhesion Molecules

##### Memory Plasma Cell Surface Markers

The phenotype of plasma cells might provide useful information about how external stimuli trigger intrinsic signals and play a role in the maintenance of memory plasma cells. The expression of CD138, TACI, BCMA, Sca-1, Ly6C, Ly6K, CD28, SLAMF7, and CD98 is a hallmark of mouse plasma cells. The human genome lacks direct homologs of murine Ly6A, Ly6C1/2, Ly6K (63). In mice, short-lived plasma cells can be distinguished from long-lived memory plasma cells by high expression of B220 (a relatively B-cell-specific isoform of CD45) and MHCII (63, 64). Human plasma cells are characterized by the co-expression of CD138 and CD38, which allows for the identification of plasma cells in the bone marrow or in cell suspensions from tissues by flow cytometry. These terminally differentiated B cells lose the ability to express CD19 and CD20 (B cell marker) on the cell surface while retaining cell surface expression of CD27. However, there is a diverse range of phenotypic markers of plasma cells (65).

CD138 (syndecan-1, Sdc-1) is a member of the syndecan family of four structurally related cell surface heparan sulfate proteoglycans (HPSGs) (66). Among non-hematopoietic cells, CD138 expression is high on epithelial cells and lower on a variety of other cell types, including endothelial cells and fibroblasts (67). Plasma cells at higher level (68) and pre-B cells at lower level (69) are the only hematopoietic cells that express CD138. High expression of CD138 on plasma cells is a hallmark of their identification, which is upregulated during differentiation from plasmablasts into plasma cells (69). CD138 is involved in many cellular functions, including cell-cell adhesion and cell-matrix adhesion (70). *In-vitro* plasma cells adhere to type I collagen of the bone marrow stromal matrix via CD138 (synecan-1) (71). Some investigators doubt that CD138 is important for plasma cell function under normal conditions (72), but recent evidence shows that CD138 plays a direct cell-intrinsic role in plasma cell survival *in vivo* (73). These authors suggest that CD138 plays a major role in protecting plasma cells from premature apoptosis by using its heparin side chains to substantially increase IL-6 and APRIL presentation to their receptors on plasma cells, leading to increased cytokine signaling and higher expression of the pro-survival proteins Bcl-2 and Mcl-1. Surface expression of CD138 on plasma cells does not impair its early differentiation or proliferation, but rather promotes or correlates with the survival of mature plasma cells.

CD38 is a type II transmembrane glycoprotein. Its extracellular domain acts as an enzyme that converts nicotinamide adenine dinucleotide (NAD^+^)/NADP^+^ into cADPR, ADP-ribose and NAADP, all of which are intracellular calcium-mobilizing agents (74). CD38 is expressed on most thymocytes, some activated peripheral blood T cells and B cells, plasma cells, and dendritic cells. Memory plasma cells express high levels of CD38 compared to their precursors (75, 76). Apparently, this CD38 molecule is distinct from CD38 on other cells because a lamprey monoclonal antibody that recognizes a unique epitope of the CD38 ectoenzyme specifically reacts with plasmablasts and plasma cells in healthy individuals and in most human multiple myelomas (77). CD38 molecules on the plasma membrane are in close contact with the BCR complex and with molecules regulating homing (CXCR4 and CD49d) (78).

CD19, a co-receptor of the BCR complex, is one of the earliest and most specific markers of B-lineage cells (79). Plasma cells in human bone marrow express CD19 in a heterogenic manner. The majority of plasma cells express CD19, but a minor group of plasma cells is CD19^neg^. There is now increasing evidence that memory plasma cells among the CD19^neg^ plasma cell population are enriched in human bone marrow (80–82). A recent study using a new staining protocol of plasma cells in mice, could also detect IgG-secreting cells with CD19^low^ B220^low^ CD138^high^ Blimp-1^high^ in bone marrow which are most likely memory plasma cells (76). Lack of CD19 expression may be considered as a candidate marker for memory plasma cells maintaining long-term memory, but its mechanism is unknown (83). Compared to CD19^pos^ plasma cells, CD19^neg^ bone marrow plasma cells have a prosurvival mature phenotype: low expression of CD95 and high expression of Bcl2 and less proliferating Ki67 cells. This is a sign of long-term stability of this subset in human bone marrow (84, 85). It has been recently demonstrated that CD19^neg^ CD45^neg^ plasma cells persist for at least two decades in the human small intestine (82). This study also has shown that CD19^neg^ plasma cells isolated from the small intestine of elderly subjects contain rotavirus-specific clones. These findings support the lifetime selection and maintenance of protective plasma cells in the human intestine (82). Therefore, CD19^neg^ plasma cells are not restricted to plasma cells in the bone marrow, but can also be detected in the gut. Of note, it has been demonstrated that CD19 loss can occur in a subset of plasmablasts at an early stage of the immune response and, thus, is not strictly dependent on plasma cell aging (79).

##### Adhesion Molecules

A variety of different adhesion molecules such as very late antigen 4 (VLA-4, integrin α4β1), lymphocyte function-associated antigen 1 (LFA-1, integrin α_L_β2), endothelial-cell selectin (E-selectin) ligand, platelet selectin (P-selectin) ligand, CD11a, CD18, CD44, and CD93 are expressed on plasma cells (20). VLA-4 and LFA-1 have a high impact on the survival of plasma cells. VLA-4 binds to VCAM-1 on stromal cells, and extracellular matrix components fibronectin and osteopontin present in the bone marrow. LFA-1 binds to three different molecules of the immunoglobulin superfamily: ICAM-1, ICAM-2, ICAM-3. Both VLA-4 and LFA-1 probably act by fixating the plasma cells in their niches. Their importance for bone marrow plasma cell survival has been demonstrated by co-blockade of LFA-1 and VLA-4 adhesion molecules *in vivo*, which resulted in a transient 75% reduction of bone marrow plasma cells in wild-type mice (86). However, the administration of integrin-blocking antibodies do not lead to strong plasma cell depletion in lupus prone mice (87). CD93 has also been suggested to promote plasma cell survival by functioning as an adhesion molecule (88) CD44 binds to hyaluronic acid, a protein of the extracellular matrix (89). CD44 itself is a surface marker of mesenchymal stem cells (90) and is involved in cell–cell and cell–extracellular matrix adhesion. Bone marrow plasma cells express high levels of CD44, which prolongs the survival of human plasma cells *in vitro* (30, 51). It has been reported that cell contact between plasma cells and stromal cells via a CD44 variant isoform induces IL-6 production by stromal cells (91).

Several other cell-surface proteins like CD28 (92) and CD37 promote various aspects of plasma cell longevity (93). CD28 expressed on bone marrow plasma cells has been shown to be essential for plasma cell longevity (50, 92), and CD28-CD80/86 interaction modulates short-lived and memory plasma cell function (94). CD28 expression triggers an intrinsic survival signal, possibly through activation of the NF-κB pathway and by upregulation of BCMA. This protection is specific to bone marrow memory plasma cells and, at least in malignant plasma cells, is mediated by CD28's engagement of CD80/86 on myeloid cells and subsequent IL-6 secretion (95). CD37, a tetraspanin protein, is essential for the clustering of VLA4 molecules on B cells necessary for activation of the Akt survival pathway. CD37-defient mice have reduced numbers of IgG secreting plasma cells compared to wild-type mice (93).

In summary, multiple molecules expressed on plasma cells contribute to plasma cell survival. The above-mentioned examples show that progress is being made, but many questions remain unanswered. Thus, more data is needed for a better understanding of the processes controlling plasma cell homing and longevity (96).

## Intracellular Factors and Mechanisms

Many intracellular factors and mechanisms are related to memory plasma cell programming. Together, they build up a complex network that controls various biological functions of memory plasma cells, including their differentiation, maintenance, and death as well as antibody synthesis and secretion. These intracellular factors and mechanisms have complicated functions and influence each other. This might explain why some studies have yielded conflicting findings and conclusions. The real pathways, especially how these intracellular factors and mechanisms communicate with the extracellular factors, are not comprehensively understood. Here, we focus on some recently described factors and mechanisms that are considered to correlate with the survival and maintenance of memory plasma cells.

### Differentiation-Related Factors

The differentiation of activated B cells into plasma cells requires coordinated expression changes in hundreds of genes. Interferon regulatory factor 4 (IRF4), B lymphocyte-induced maturation protein 1 (Blimp-1), and X-box-binding protein 1 (XBP-1) are the three most important transcription factors guiding the plasma cell development program (17, 97). IRF4 is required for class switch recombination, germinal center (GC) B cell formation, and plasma cell differentiation (98–100). IRF4 functions are dose-dependent. Low levels of IRF4 or even transient induction of IRF4 is sufficient to induce GC B-cell formation, while high concentrations of IRF4 promote the generation of plasma cells and antagonize the GC fate by repressing *Bcl6* and by activating both Blimp1 and Zbtb20 (zinc finger and BTB domain-containing protein 20) (17, 100, 101). It has been shown that plasma cells residing in murine bone marrow disappeared immediately after conditional inactivation of *Irf4*, and that the effect can last for the whole observation time period of several weeks (102). Therefore, in addition to the defects in GC B-cell formation (100) and plasma cell differentiation caused by the loss of IRF4, the available results indicate that IRF4 plays an essential part in memory plasma cell survival, potentially by regulating some key survival molecules, such as myeloid cell leukemia 1 (Mcl-1) (102).

Blimp-1 is a transcriptional “master regulator” that is necessary for plasma cell differentiation (103, 104). During the B cell to plasma cell transition, 648 genes are upregulated and 424 are downregulated. Blimp-1 activates 38% (245) of these upregulated genes and represses 41% (105) of the downregulated ones (106). It directly regulates several transcription factors and important gene programs to facilitate the post-mitotic state of mature plasma cells (17, 107, 108). Within the B cell lineage, Blimp-1 is exclusively expressed in plasma cells, and its expression is higher in mature memory plasma cells than in short-lived plasma cells (plasmablasts) (109). By using a conditionally Blimp-1 deficient mouse model, it has been shown that Blimp-1 is required for the maintenance of memory plasma cells in the bone marrow and for the long-term maintenance of antigen-specific immunoglobulin in serum. In this mouse model, the number of memory plasma cells in the bone marrow decreases 4-fold, resulting in a drop in antigen-specific IgG1 levels in serum 3 to 4 weeks after inactivation of *Prdm1*, which encodes Blimp-1 (110). However, by using a GFP reporter mouse model to track plasma cells at higher resolution, another study more recently has demonstrated that plasma cell numbers in the bone marrow and spleen remain stable for many weeks in spite of a lack of Blimp-1, although the Blimp-1 deficient plasma cells lost their ability to secrete antibodies (102). Similar results are obtained after transferring B cells from these mice into B- and T-cell-deficient Rag1^−/−^ mice after conditional Blimp-1 inactivation. Furthermore, this study suggests that Blimp-1 is essential for the establishment of the full plasma cell transcriptome but that once it has been established, plasma cell identity is maintained independently of Blimp-1 (102).

### Endoplasmic Reticulum Stress-Related Factors

Memory plasma cells continuously secrete antibodies which allow the immune system to maintain a stable humoral immunological memory over long periods (8). To maintain stable levels in serum, one plasma cell secrets about 10^3^ antibodies per second, approximately 2 ng per day (111, 112). To maintain this large-scale and stable antibody synthesis and secretory capacity, plasma cells require a specialized machinery, and metabolic activity. The endoplasmic reticulum (ER) is the major organelle for the synthesis and folding of secreted and transmembrane proteins. Plasma cells have continuous ER stress. When protein-folding requirements exceed the processing capacity of the ER, the accumulated misfolded and unfolded proteins trigger the unfolded protein response (UPR), resulting in the adjustment of protein synthesis and the enhancement of ER folding capacity as well as increased degradation of misfolded proteins and enhanced ER biogenesis (113, 114). However, when these attempts fail and ER stress is unabated, UPR signaling typically switches to a pro-apoptotic mode known as the terminal UPR (115). The pro-apoptotic factor Chop (C/EBP homologous protein) is a characteristic marker for terminal UPR-induced apoptosis.

There are three regulatory arms of the UPR: PERK (protein kinase RNA activated (PKR)-like ER kinase), ATF6α (activating transcription factor 6α), and IRE1 (inositol-requiring enzyme-1) (113). Although mature B cells express high levels of PERK and ATF6α, physiologically, both PERK and ATF6α are dispensable for plasma cell differentiation, immunoglobulin secretion and survival (116, 117). Blimp-1 is intimately involved in the UPR. It directly regulates *Atf6* and 38% of the downstream genes of the UPR (102). As a component of the IRE1 branch, XBP-1 is an important transcription factor associated to UPR, which induces the transcription of a wide variety of ER-resident molecular chaperones and protein-folding enzymes that work together to increase ER size and function (118). Additionally, the induction of *Xbp1*, which is downstream of Blimp-1, is required for this marked ER expansion and increased protein synthesis (119). XBP-1 is required for the generation of plasma cells. In XBP-1 deficient mice, immunoglobulin levels are low and plasma cells are notably absent (120). However, later studies suggest that XBP-1 is required more specifically for immunoglobulin production (121–123). Conditional inactivation of *Xbp1* has no effect on the size of the plasma cell population, while XBP-1 deficiency in bone marrow plasma cells results in a global decrease in immunoglobulin transcripts and protein expression which correlates with reduced immunoglobulin secretion (102), but does not have a direct effect on the maintenance of memory plasma cells. Based on the current evidence, it seems that the three main arms of UPR do not directly influence the survival and longevity of memory plasma cell.

The inducible nitric oxide synthase (iNOS), which can be induced by XBP-1 (124), is associated with various mammalian physiology functions, including ER stress. iNOS has been found to modulate components of the UPR. Several mRNA levels related to ER stress are significantly lower in iNOS-deficient plasma cells (125). Both iNOS deficiency and iNOS inhibitor treatments cause plasma cells to have shorter life spans *in vitro* and *in vivo*. Bone marrow memory plasma cell numbers are significantly lowered in iNOS-deficient mice and wild-type mice treated with an iNOS inhibitor, and this decrease is accompanied by a significant decrease in the levels of antigen-specific antibodies. The effect of iNOS on the ER suggests that it has an effect on plasma cell survival. The finding that iNOS is also required for plasma cell responses to IL-6 and APRIL suggests an additional contribution of iNOS to the maintenance of memory plasma cells (125, 126).

Another system linked to ER stress is the ubiquitin-proteasome system, which is responsible for the degradation of not needed and misfolded proteins inside the cell. Bortezomib can inhibit the proteasome function and induce the efficient depletion of plasma cells, including memory plasma cells, in lupus mice (127). After bortezomib treatment, mRNA levels of Chop, a characteristic marker for the terminal UPR, increase 40-fold in splenic plasma cells and 20-fold in bone marrow plasma cells, resulting in the induction of terminal UPR and cell death. Another mechanism contributing to bortezomib-induced cell death is the inhibition of anti-apoptotic transcription factor NF-kB activity (127).

### Autophagy

As misfolded proteins accumulate in the ER, autophagy functions as a crucial adaptive “self-eating” process by which autophagosomes envelop and degrade cellular components, and thus ameliorate ER stress. Similarly to the unfolded protein response, autophagy can result in either cell survival or cell death (114). Autophagy is a catabolic process related to lysosomal activity. There are three major types of autophagy: macroautophagy, chaperone-mediated autophagy (CMA), and microautophagy. *Microautophagy* involves direct invagination of the lysosomal membrane. C*haperone-mediated autophagy* involves the direct translocation of proteins into lysosomes. *Macroautophagy* leads to the integration of cytoplasmic material into vesicles that ultimately fuse with lysosomes; it is central to lymphocyte homeostasis, which is under the control of autophagy-related gene (ATG) products (128). Using mice in which *Atg5* is conditionally deleted in B lymphocytes, antibody responses are significantly diminished during antigen-specific immunization, parasitic infection, and mucosal inflammation (129). Moreover, *Atg5*-deficient B cells retain the ability to produce immunoglobulin and undergo class-switch recombination, but are impaired in their ability to terminally differentiate into plasma cells and, therefore, are unable to mount an effective antibody response, since the total plasma cell numbers in spleen and mesenteric lymph nodes are significantly low after immunization (129). However, another study with *Atg5*-deficient mouse model has shown that the antigen-specific plasma cell number in spleen is similar to the control group 14 days after immunization (130). Memory plasma cells from the bone marrow have higher autophagic activity than B cells. *Atg5*-deficient plasma cells have a larger ER and more ER stress signaling, which leads to higher expression of Blimp-1 and immunoglobulins, and to increased antibody secretion (130). The enhanced immunoglobulin synthesis is associated with more death of mutant plasma cells. The immunized *Atg5*-deficient mice have normal GC responses, but a 90% reduction of antigen-specific bone marrow memory plasma cells, demonstrating that Atg5 is specifically required for the maintenance of bone marrow memory plasma cells (130). Similarly, the *in vitro* study has shown that a lack of autophagy causes a substantial increase in the death of murine plasma cells and that 1 year after immunization, Atg5-deficient mice have remarkably fewer antigen-specific memory plasma cells in the bone marrow than wild-type mice (131). Another study using a murine autophagy-deficient autoimmune model has revealed that a decrease in memory plasma cells in the bone marrow is accompanied by a decrease in serum anti-dsDNA IgG antibody levels (132). These findings confirm that autophagy is important for the maintenance of memory plasma cells. So far, the contribution of other autophagy factors is not known.

### Metabolism

As they require the secretion of large quantities of glycosylated antibodies, which consumes 90% of their glucose utilization, plasma cells have high metabolic and energy pressure. Human and murine memory plasma cells can robustly engage pyruvate-dependent respiration and take up more glucose, which is essential for the generation of pyruvate. Targeting mitochondrial pyruvate carriers Mpc1 and Mpc2 *in vitro* reduces the survival of memory plasma cells significantly (133). The conditional *Mpc2*-deficient mice result in a significant loss of bone marrow memory plasma cells and a corresponding reduction of antigen-specific antibody titers in serum. These findings suggest that glucose uptake and mitochondrial pyruvate import promote the long-term persistence of memory plasma cells (133, 134). Interestingly, glucose can stabilize the expression of Mcl-1 (135, 136), which is essential for the survival of memory plasma cells (see below). Other nutrients besides glucose also contribute to plasma cell functions. Amino acids are the basis for antibody synthesis. Expression of CD98, a common subunit of many amino acid transporters and thus a marker of amino acid availability, is controlled by the transcription factor Blimp-1 and is very highly expressed in plasma cells, especially memory plasma cells (64, 102, 117). CD98 deficiency leads to severe antibody defects, and autophagy contributes to the metabolism of amino acids as cellular components are recycled, whereby the autophagy activity is higher in memory plasma cells than in plasmablasts (117). The metabolism of short-chain fatty acids (SCFAs) produced by gut microbiota are involved in plasma cell differentiation and promote antigen-specific host antibody responses (137).

Ectonucleotide pyrophosphatase/phosphodiesterase 1 (ENPP1), first identified as a membrane alloantigen, is involved in ATP-derived energy production. ENPP1 expression gradually increases during B cell differentiation to plasma cells, and bone marrow plasma cells show higher ENPP1 expression than their splenic counterparts in both mice and humans. Furthermore, bone marrow memory plasma cells express about 2-fold more ENPP1 than plasmablasts (138). ENPP1 deficiency does not affect GC formation or plasmablast migration. However, plasma cells residing in the bone marrow of *Enpp1*^−/−^ mice take up less glucose and the frequency of antigen-specific memory plasma cells is significantly lower in the bone marrow than wild-type controls (138). ENPP1-deficient plasma cells have an impaired glycolysis pathway, which leads to reduced levels of energy production. Considering the 2-fold higher ENPP1 expression in bone marrow memory plasma cells, it suggests that ENPP1 allows bone marrow memory plasma cells to consume more glucose in order to better fuel higher antibody production levels and longer survival times (138).

### Anti-apoptotic Factors

The NF-κB family of transcription factors governs the expression of multiple genes involved in cell survival, proliferation and effector functions. The primary contribution of NF-κB to lymphocytes is to assure cell survival. The anti-apoptotic functions of NF-κB are crucial for lymphocytes, even after they become mature (139). As NF-κB is involved in activities such as proliferation, activation, and GC formation during B cell differentiation (140–142), it influences the differentiation of plasma cells. Several factors like BAFF-BAFF-R axis and CD40-CD40L axis have been shown to activate the NF-κB pathway and mediate B and plasma cell differentiation. Adhesion molecules such as ICAM-1 and VCAM-1 are also regulated by NF-κB signaling (143). These factors are involved in the construction of survival niches for memory plasma cells, probably indirectly, through the NF-κB pathway.

Ras-related in brain 7 (Rab7) inhibition and knock-out studies provide further evidence of the role of the NF-κB pathway in the maintenance of memory plasma cells. Rab7 is a small GTPase that plays a B cell–intrinsic role in antibody response and promotes class-switch recombination by mediating NF-κB activation (144). One study has showed that Rab7 activity inhibition or Rab7 gene knockout results in reduced numbers of plasma cells, including memory plasma cells, and that it consequently suppresses IgG anti-dsDNA autoantibody responses, prevents the development of disease symptoms, and extends the lifespan of lupus mice (145); Rab7 also decreases the expression of several genes associated with memory plasma cells survival, including *Cxcr4, Irf4, Mcl1*, and *Atg5*, but not *Prdm1* and *Xbp1*. Interestingly, the apoptosis of cultured CD19^−^CD138^hi^ plasma cells induced by Rab7 inhibition can be prevented by enforced NF-κB activation (145). In another study, the treatment of lupus mice with resveratrol, a small polyphenol anti-inflammatory agent, enhances the expression of FcγRIIB on B cells and plasma cells, resulting in a marked depletion of plasma cells in the spleen and bone marrow, thereby decreasing serum autoantibody titers and ameliorating lupus nephritis; the authors have concluded that this upregulation of FcγRIIB is NF-κB dependent (146).

Bcl-2, Bcl-xL, and Mcl-1 are anti-apoptotic members of the Bcl-2 family expressed on plasma cells (56, 147). Various studies have shown that Bcl-2 and Bcl-xL are involved in plasma cell differentiation (148, 149), but the presence of both is not crucial for the survival of existing plasma cells (56, 150). Mcl-1 expression, regulated by the BCMA, is higher in bone marrow plasma cells than in plasma cells residing in other lymphoid organs (56). BCMA is a receptor for APRIL, which is an important survival factor for memory plasma cells, as described above. BCMA is an essential factor for the survival of memory plasma cells in the bone marrow. BCMA^−/−^ mice have a 20% decrease in plasma cells in the bone marrow compared to their wild-type counterparts (57). The enzyme γ-secretase directly cleaves BCMA and releases soluble BCMA, which acts as a decoy that neutralizes APRIL. The inhibition of γ-secretase *in vivo* enhances BCMA surface expression in plasma cells and increases their number in the bone marrow (151). Another study confirms the importance of the APRIL-BCMA axis in plasma cell survival in the bone marrow and indicates that this process requires the transcriptional induction of Mcl1 (56). After deletion of Mcl1, the percentage and absolute numbers of total plasma cells and antigen-specific plasma cells are significantly lower than in wild-type mice, which highlights the important role of this APRIL/BCMA/Mcl-1 signaling pathway for the long-term maintenance of memory plasma cells. The study from a mouse plasma cell line has shown that Blimp-1 can positively regulate the expression of the BCMA gene (152). Although the induction of BCMA is part of a transcriptional program during plasma cell differentiation, however, investigations in Blimp-1 deficient GFP reporter mice have indicated that the BCMA-mediated plasma cell survival pathway is independent of Blimp-1 (56). So far, this APRIL/BCMA/Mcl-1 pathway appears to be the best-characterized survival pathway of memory plasma cells (96).

### MicroRNAs

MicroRNAs (miRNAs) are small, non-coding RNA molecules (containing about 20–22 nucleotides) that post-transcriptionally regulate gene expression in plants and metazoans. Until now, about 2,500 human and 1,900 mouse miRNAs which are functionally involved in most physiological cellular processes, including proliferation, development, and differentiation, have been identified (153–155). Many miRNAs are involved in B cell and plasma cell biology, for example, miR-30, miR-217, miR-28, miR-150, miR-155, miR-361, miR-125b, miR-181b, miR-21, miR-24-3p, miR-148a, and miR-17-92, etc. (156). MicroRNA-150 is specifically expressed in mature lymphocytes; it directly targets the transcription factor c-Myb (157), which is required for newly generated plasma cells migrating toward CXCL12 and therefore regulates the establishment of the memory plasma cell pool (158). MicroRNA-155 is required for the B-cell response to antigens. In miR-155-deficient mice, the number of GC B cells is reduced. B cells lacking miR-155 show a reduced GC response and failed secretion of class-switched, high-affinity IgG1 antibodies (159, 160). MicroRNA-125b regulates GC B-cell responses by targeting transcription factors IRF-4 and Blimp-1, and thereby inhibiting plasma cell differentiation (161). MicroRNA-24–3p has been identified as a direct mediator of human plasma cell survival, which is upregulated by IL-6 and CXCL12. Under induced ER stress, the upregulation of miR-24-3p expression by IL-6 can rescue plasma cells from apoptosis through the mitogen-activated protein kinase pathway (162). MicroRNA-148a can protect immature B cells from apoptosis and regulate B cell tolerance; it is upregulated in lymphocytes from lupus patients and lupus mice and accelerates the development of autoimmunity (163). It has been shown that miR-148a is upregulated in activated naive murine B cells, that it is the most abundant miRNA in memory plasma cells in both humans and mice, and that it promotes plasmablast differentiation and survival *in vitro* (164). A significant decrease in serum antibody levels and plasma cell numbers has been observed in conditional B-cell-specific miR-148a knockout mice with and without immunization, and in tamoxifen-inducible miR-148a-deficient mouse, the number of bone marrow memory plasma cells is significantly reduced, suggesting that miR-148a controls the differentiation of B cells into plasmablasts and the survival of memory plasma cells (165).

### Other Factors

Zbtb20 is a broad complex, tramtrack, bric-à-brac, and zinc finger (BTB-ZF) protein expressed in GC B cells; it is upregulated during plasma cell differentiation and is highly expressed in memory plasma cells in an IRF4-dependent manner. Zbtb20 conditional knockout mice are characterized by a blunted antibody response and a significant loss of plasma cells in the bone marrow; these findings indicate that Zbtb20 is essential for the maintenance of memory plasma cells in the bone marrow and for the persistence of antigen-specific immunoglobulin levels in serum (101). In this study the expressions of *Bcma* and *Mcl1* in plasma cells are similar in knockout and wild-type mice (as determined by quantitative RT-PCR), whereas another study in Zbtb20-deficient mice demonstrates the reduced expression of *Mcl1* in bone marrow plasma cells (as analyzed by single-cell quantitative RT-PCR), suggesting that Zbtb20 may be required for the maximal expression of Mcl-1 (166). Interestingly, the requirement for Zbtb20 appears to be dependent on the type of adjuvant used. After alum-adjuvanted immunization, antigen-specific memory plasma cells fail to accumulate in the bone marrow, leading to a progressive loss of antibody production, whereas adjuvants that activate TLR2 and TLR4 restore long-term antibody production by inducing compensatory survival pathways in the plasma cells of Zbtb20-deficient mice (166).

Another regulator expressed in plasma cells is the tyrosine kinase Lyn, a negative regulator for many signaling pathways. Lyn attenuates signal transducer and activator of transcription 3 (STAT3) signaling, which can mediate the upregulation of Blimp-1 during plasma cell differentiation (167), of STAT3 responses to IL-6, and of the IL-6/JAK/STAT3 pathway, therefore supporting plasma cell survival and immunoglobulin secretion (168). A study in Lyn-deficient mice has showed that, in the absence of Lyn, memory plasma cells accumulate and have improved survival, and that the expression of CXCR4 on plasma cells is enhanced. Furthermore, cultured Lyn-deficient plasma cells show better *in vitro* survival with IL-6 but not with APRIL, indicating that Lyn regulates the survival of memory plasma cells through the IL-6/STAT3 pathway (169).

**Figure 1:**
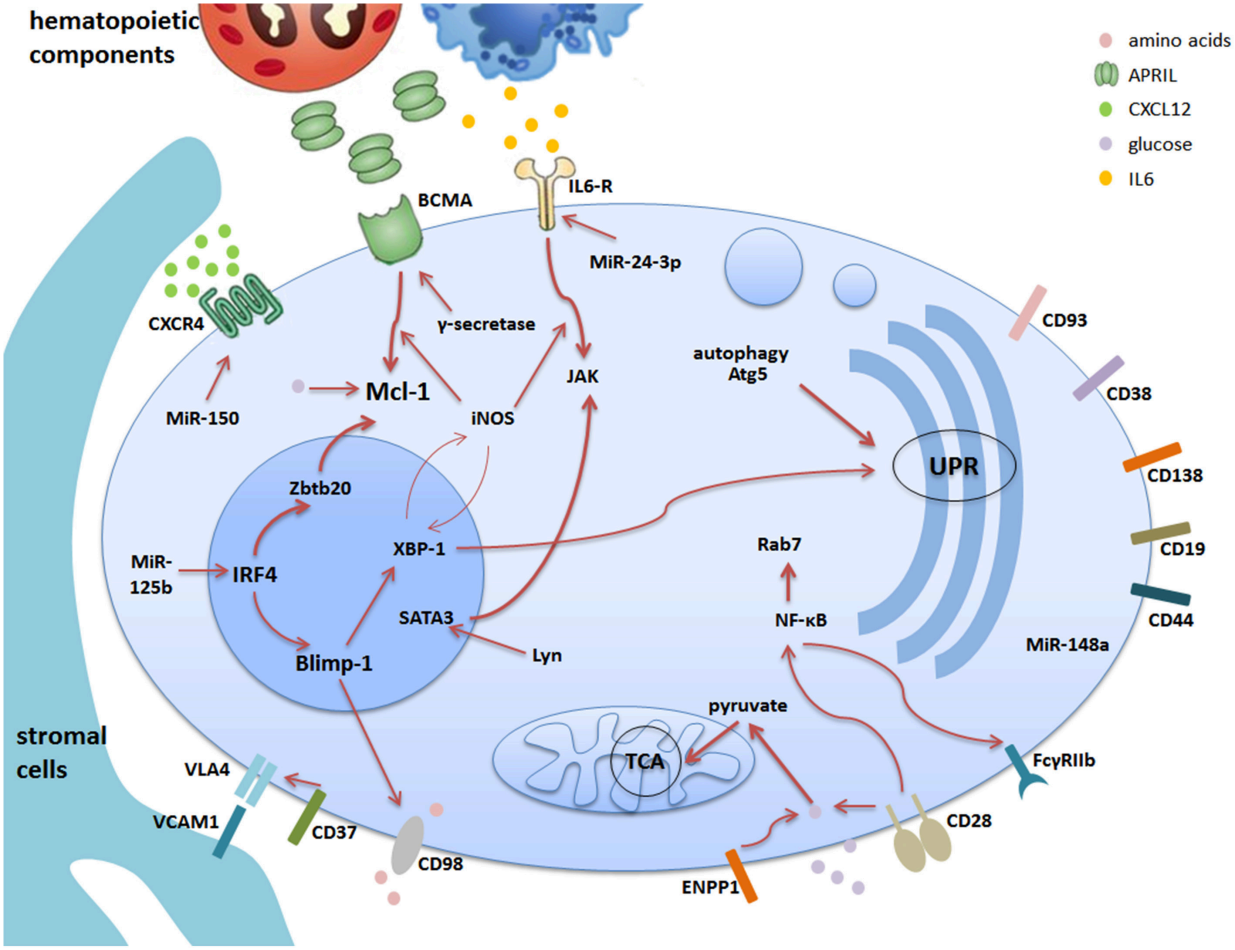
The survival network for memory plasma cells in the bone marrow. Bone marrow is the major site of memory plasma cells. The stromal cells together with hematopoietic cells construct the microenvironment supporting the survival of memory plasma cells via cell-contact or secretion of soluble factors. Different plasma cell surface molecules are communicating with intracellular factors via different pathways regulated by internal factors and mechanisms. This cartoon summarizes the current understanding of the complex network consisting of stromal and hematopoietic cells, soluble factors, receptors and signaling pathways. The thicker arrows indicate pathways, which are key players in the maintenance of memory plasma cells.

The lifestyle of memory plasma cell is complex. It involves many intracellular factors and mechanisms, all of which influence the maintenance of memory plasma cells more or less. Apparently, communication between the different factors and mechanisms is essential for establishing a survival network for memory plasma cells (Table 1; Figure 1). However, the signaling pathways, especially the patterns of connecting extracellular factors, are still bewildering and need to be further investigated.

**Table 1:** Extrinsic and intrinsic factors contributing to the maintenance of memory plasma cells.

**External factors**		**Internal factors and mechanisms**
**Cellular compartments**	**Molecular compartments**	➢ Differentiation related Factors • IRF4 • BLIMP-1 • XBP-1 ➢ Endoplasmic reticulum (ER) stress related factors and mechanism • UPR • iNOS • Ubiquiton-proteasome response ➢ Autophagy and metabolism •Anti-apoptosis factors • NF-kB family • Bcl-2 Family ➢ Others • Zbtb20 • Tyrosine Kinase Lyn • MicroRNA
➢ Stromal Cells (mesenchymal origin) • CXCL12 + cells ➢ Hematopoietic-origin cells • Megakaryocytes • Basophils • Dendritic cells • Monocytes/Macrophages • Neutrophils • Eosinophils • Regulatory T cells	➢ Soluble Factors • Cytokines ° TNF-superfamily (APRIL, BAFF, TNF-a) ° IL-6 • Chemokines ° CXCL-12 ➢ Membrane-bound Factors • Plasma cell (PCs) surface markers ° CD138 ° CD38 ° CD19 • Adhesion molecules expressed on PCs ° VLA-4 ° LFA-1 ° CD93 ° CD44 ° CD28 ° CD37	

## Maintenance of Memory Plasma Cells in Inflamed Tissues

The bone marrow is the primary survival niche for memory plasma cells. In addition, secondary lymphoid organs like the spleen provide a limited number of plasma cell survival niches. However, in the presence of chronic inflammation, memory plasma cells can also be detected in inflamed tissues (20, 170, 171), such as kidneys (105, 172–176), central nervous system (177–179), lungs (180, 181), nose (182), lymph nodes (183), salivary glands (184, 185), joints (186, 187), and tonsils (188, 189). The infiltration of local plasma cells appears to be associated with the severity of the inflammatory disease. Little is known about how the memory plasma cells are maintained in the inflamed tissues and what kinds of survival niches support them. In this section, we will review recent findings regarding the presence of memory plasma cells in inflamed tissues.

### CXCL12 Axis

The CXCL12/CXCR4 axis supports the maintenance of memory plasma cells in the bone marrow, as described above. In lupus prone mice (NZB/W), CXCL12 expression is higher than in healthy mice. Various studies have shown that CXCL12 levels are elevated in the kidneys of older mice with nephritis (174, 190, 191), and that neutralization of CXCL12 with a monoclonal antibody at an early age can prevent the development of proteinuria and prolong the survival (190). Treatment with AMD3100, a CXCR4 blocker, significantly decreases the number of memory plasma cells in the kidneys of NZB/W mice (175). Similar results have been achieved by inhibiting CXCR4 with the antagonist CTCE-9908 in another murine lupus model (191). About 60% of plasma cells in inflamed NZB/W kidneys are in contact with CD11b^+^ macrophage-like cells (173), which are the prime source of CXCL12 (192). Using a collagen-induced arthritis model, it has been observed that CXCL12 expression is increased in the inflamed joints, and that treatment with the CXCR4 blocker AMD3100 provides clinical benefits (193). In rheumatoid arthritis (RA), the expression of CXCL12 in synovial tissues is increased (194, 195), and CXCL12 seems to be predominantly expressed by endothelial venules and synoviocytes in the synovial tissues (196–199). These findings resemble the pictures of the CXCL12-expressing stromal cells organizing the survival niches for memory plasma cells in the bone marrow (170).

In a murine model of induced experimental autoimmune encephalomyelitis (EAE), an upregulation of CXCL12 has been detected in the inflamed spinal cord where memory plasma cells are localized (179). Similar co-localization of memory plasma cells with CXCL12-expressing epithelial and infiltrating mononuclear cells has been detected in the salivary glands of patients with primary Sjögren's syndrome (184). However, in a hepatitis virus-induced central nervous system inflammation mouse model, virus-specific IgM and IgG antibodies are detected in the spinal cord, and plasma cells are predominantly found in demyelinated lesions and adjacent white matter (178). While, mRNA levels of CXCL12 do not always exceed baseline levels throughout this inflammatory condition (178).

### APRIL/BAFF Axis

APRIL and BAFF expression levels are higher in the inflamed kidneys of NZB/W mice (174). In human lupus nephritis, elevated mRNA levels of APRIL and BAFF have been detected in renal biopsies from patients refractory to immunosuppressive therapies (200). In RA, BCMA and APRIL expression levels are higher in synovial fluid (187, 201), and the APRIL levels closely correlate with the local plasma cell counts. The main source of APRIL is infiltrating neutrophils and CD68+ macrophages, and CD138+ plasma cells are found to be in tight contact with CD68+ macrophages in the zones of high concentration of secreted APRIL (202). Furthermore, APRIL and BAFF released by microglia and astrocytes in the spinal cord are present in the same area in which memory plasma cells are found. Notably, the memory plasma cells are strongly positive for both APRIL and BAFF, which suggests that a self-sufficiency mode may exist under inflammatory and autoimmune disease conditions (179). Moreover, the mRNA levels of BCMA, TACI, APRIL and BAFF in the spinal cord are increased during inflammation, indicating that these factors may support the survival of local plasma cells (178).

In inflamed nasal tissues from patients with granulomatosis with polyangiitis (GPA), plasma cells are found in close proximity to APRIL secreted by macrophages, giant cells, and epithelial cells. (182). In allergen-challenged mice, BAFF levels are significantly increased in lung and bronchoalveolar lavage fluid (203), and the number of eosinophils is increased and associated with infection and allergic diseases. Although eosinophils have been shown to support the maintenance of memory plasma cells in the bone marrow and of IgA+ plasma cells in the intestine (44), the number of IgA+ plasma cells is not reduced in the lungs of eosinophil-deficient mice (204).

### Others Factors

Under inflammatory conditions, plasma cells are detectable in the inflamed joints of patients with RA and osteoarthritis (186, 187, 205, 206). Many factors like ICAM1, VCAM1, VLA-4, and IL-6 are increased in inflamed synovial tissues (207–209), which may build up the microenvironment needed for the maintenance of local plasma cells. Other types of cells may also contribute to the establishment of survival niches in inflamed joints. In RA, nurse-like cells produce enhanced levels of many cytokines (e.g., IL-6) and promote B cell survival and differentiation. Interestingly, transmission electron microscopy analysis of RA synovial tissues has shown numerous plasma cells are surrounded by synovial long slender cytoplasmic dendritic cells with spines or finger-like protrusions, which cell membrane appears to be fused or very tightly attached to the cell membrane of the paired plasma cell (210). Immunofluorescence staining studies also show that both CD14+ dendritic cells and CD138+ plasma cells in synovial tissue reside in close proximity (211, 212).

In an adapted multiple sclerosis murine model of EAE, VCAM-1 is upregulated; memory plasma cells are localized in areas of increased VCAM-1 expression (179). Further research has shown that, in the inflamed salivary glands of patients with Sjögren's syndrome, Ki67 negative memory plasma cells are tightly juxtaposed to the ductal and acinar epithelia, which highly express IL-6 (184).

Plasma cells are also detectable in lung tissues during allergic airway inflammation. Nerve growth factor (NGF) and neurotrophin-3, mainly secreted by local T cells and macrophages, appear to support the survival of these plasma cells, which express neurotrophin receptors due to upregulation of the anti-apoptotic protein Bcl2. One study has showed that inhibition of neurotrophin receptors significantly reduces local plasma cell numbers and serum antibody levels, and that overexpression of NGF results in higher plasma cell counts in the perialveolar area. These data suggest that NGF might be essential for the local survival for plasma cells (213). In patients with chronic bronchitis and obstructive pulmonary disease, plasma cells are particularly abundant in the subepithelium and interstitium between submucosal gland acini, where they are co-localized with IL-4-positive cells. The latter cells are identified as CD68+ monocytes/macrophages and CD20+ B cells and, interestingly, over 60% of the plasma cells themselves express IL-4 in these inflamed tissues (214).

So far, investigators who have tried to explain the survival mechanisms for memory plasma cells under inflammatory conditions based their studies on the assumption that the memory plasma cell environment in inflamed tissues is like that in the bone marrow, but if this is actually so in reality is still unclear. Current findings suggest that the maintenance of plasma cells in inflamed tissues is supported by inflammatory cells (Table 2). In addition, plasma cells themselves are able to secrete their own survival factors such as APRIL, BAFF, and IL-6. Transcriptome comparisons of bone marrow memory plasma cells and splenic plasmablasts revealed more than 900 differentially expressed transcripts between these two types of plasma cells (117). Taking into account the different behavior of memory plasma cells in inflamed tissue, there must be differences between the memory plasma cells in bone marrow and those in inflamed tissues, healthy memory plasma cells, and their autoreactive counterparts. A study comparing the transcriptomes of these plasma cells would help to understand the survival mechanisms of (autoreactive) memory plasma cells under inflammatory conditions.

**Table 2:** Contributors to the survival of memory plasma cells in inflamed tissues.

**Inflammation**	**Factors**	**Local sources**
Kidney	CXCL12 APRIL, BAFF	Macrophages Not clearly defined
Joint	CXCL12 APRIL IL-6 Cell-cell contact ICAM1, VCAM1, VLA-4	Endothelial cells, synoviocytes Infiltrating neutrophils, macrophages Nurse-like cells Dendritic cells Not clearly defined
CNS	APRIL, BAFF CXCL12, VCAM-1	Microglia and astrocytes, plasma cells Not clearly defined
Salivary gland	CXCL12 IL-6	Epithelial infiltrating mononuclear cells Ductal and acinar epithelia
Nose	APRIL	Macrophages, giant cells, and epithelial cells
Lung	NGF, neurotrophin-3 IL-4 BAFF	Local T cells, macrophages Monocytes/macrophages, B cells, plasma cells Not clearly defined

## Conclusion

Due to their longevity, memory plasma cells residing in the bone marrow are crucial for maintaining humoral immunity independently of memory B cells, T cell help, and antigen presentation. A stable immune memory provides long-term protection against pathogens. The survival of memory plasma cells is supported by a physiological survival niche established by different cells and extra- and intra-cellular factors and mechanisms. Upsetting the niche environment will disturb the survival of memory plasma cells and, ultimately, humoral immune memory. More information on the maintenance of memory plasma cells may improve vaccination strategies.

However, if immune tolerance fails, autoreactive plasma cells can be generated and become memory plasma cells surviving in bone marrow and chronically inflamed tissues where they contribute to autoimmune pathology. Under these disease conditions, memory plasma cells are considered to be therapeutic targets (170, 172, 215). Compared to plasmablasts, memory plasma cells are resistant to most conventional therapies, including conventional immunosuppression and B cell depletion as such. This makes it clinically challenging to target memory plasma cells (87, 171, 216, 217). At present, only a few approaches are able to target memory plasma cells efficiently. Since these methods do not distinguish between memory plasma cells secreting protective and pathogenic antibodies, there is a need to develop strategies that selectively targets the pathogenic cells without affecting the protective humoral memory. Therefore, a better understanding of the lifestyle of memory plasma cells will give us clues for developing new approaches to target these cells. Although many advances have been made in research on the survival and maintenance of memory plasma cells, as we have described in this review, the path to truth is still covered with a veil that needs to be lifted in further investigations in the future.

## Author Contributions

All authors listed have made a substantial, direct and intellectual contribution to the work, and approved it for publication.

### Conflict of Interest Statement

The authors declare that the research was conducted in the absence of any commercial or financial relationships that could be construed as a potential conflict of interest.
